# From J. Smith Dodge

**Published:** 1884-04

**Authors:** J. Smith Dodge

**Affiliations:** No. 15 West 20th St., New York


No. 15 West 20th St.,

New York, March 15, 1884.

*Editor Independent Practitioner:*

Dentists have often complained of popular ignorance concerning the teeth, and have taken pains to educate the public. But I discovered the other day that the schoolmaster is abroad with all the modern improvements, and we may presently be going to school to our patients. It was in this wise: An uncommonly bright school girl of twelve or fourteen years, having just left my chair, looked into my face with her eyes full of a sweet seriousness and asked, "Doctor, have I such teeth as bacteria readily devour?" And certain symptoms of amusement on my part were not received with favor. Very truly yours,

J. Smith Dodge, D. D. S.

